# Substantial and Reproducible Individual Variability in Skeletal Muscle Outcomes in the Cross-Over Designed Planica Bed Rest Program

**DOI:** 10.3389/fphys.2021.676501

**Published:** 2021-07-16

**Authors:** Rodrigo Fernandez-Gonzalo, Adam C. McDonnell, Elizabeth J. Simpson, Ian A. Macdonald, Eric Rullman, Igor B. Mekjavic

**Affiliations:** ^1^Department of Laboratory Medicine, Division of Clinical Physiology, Karolinska Institutet, and Unit of Clinical Physiology, Karolinska University Hospital, Stockholm, Sweden; ^2^Department of Automation, Biocybernetics and Robotics, Jozef Stefan Institute, Ljubljana, Slovenia; ^3^MRC Arthritis Research UK Centre for Musculoskeletal Ageing Research, School of Life Sciences, University of Nottingham Medical School, Nottingham, United Kingdom; ^4^Department of Biomedical Physiology and Kinesiology, Simon Fraser University, Burnaby, BC, Canada

**Keywords:** FemHab, LunHab, PlanHab, skeletal muscle atrophy, microgravity

## Abstract

To evaluate the individual responses in skeletal muscle outcomes following bed rest, data from three studies (21-day PlanHab; 10-day FemHab and LunHab) were combined. Subjects (*n* = 35) participated in three cross-over campaigns within each study: normoxic (NBR) and hypoxic bed rest (HBR), and hypoxic ambulation (HAMB; used as control). Individual variability (SD_IR_) was investigated as √(SD${}_{\text{Exp}}^{2}$–SD${}_{\text{Con}}^{2}$), where SD_Exp_ and SD_Con_ are the standard deviations of the change score (i.e., post – pre) in the experimental (NBR and HBR) and the control (HAMB) groups, respectively. Repeatability and moderators of the individual variability were explored. Significant SD_IR_ was detected for knee extension torque, and thigh and calf muscle area, which translated into an individual response ranging from 3 to −17% for knee extension torque, −2 to −12% for calf muscle area, and −1 to −8% for thigh muscle area. Strong correlations were found for changes in NBR vs. HBR (i.e., repeatability) in thigh and calf muscle area (*r* = 0.65–0.75, *P* < 0.0001). Change-scores in knee extension torque, and thigh and calf muscle area strongly correlated with baseline values (*P* < 0.001; *r* between −0.5 and −0.9). Orthogonal partial least squares regression analysis explored if changes in the investigated variables could predict calf muscle area alterations. This analysis indicated that 43% of the variance in calf muscle area could be attributed to changes in all of the other variables. This is the first study using a validated methodology to report clinically relevant individual variability after bed rest in knee extension torque, calf muscle area, and (to a lower extent) thigh muscle area. Baseline values emerged as a moderator of the individual response, and a global bed rest signature served as a moderately strong predictor of the individual variation in calf muscle area alterations.

## Introduction

Interindividual differences in the physiological responses to an intervention (e.g., exercise or drugs) have received great research attention in the last decades with the aim to identify “responders” and “non-responders,” to explore the mechanisms that influence the individual responsiveness, and to promote “personalized medicine” (Mann et al., 2014; Hecksteden et al., 2015; Ross et al., 2019). However, some of the approaches used to analyze individual variability have not taken into account the variability explained by technical and/or random errors, and thus have not reported biological variability alone (Atkinson and Batterham, 2015). Hence, controlling for these factors is essential for the accurate determination of the true individual response to an intervention (Atkinson and Batterham, 2015; Atkinson et al., 2019). To tackle these limitations, the inclusion of a control group and a sufficiently large sample size to analyze variance rather than effect-sizes is crucial (Atkinson and Batterham, 2015; Hopkins, 2015). In addition, the use of cross-over designs with complete wash-out periods between intervention/control stages can be very helpful, since they offer the advantage of controlling for genetic factors influencing the individual response. It follows that only when an individual response is confirmed, potential factors (i.e., moderators or mediators) that may influence the observed individual response can be explored.

Bed rest is the gold-standard spaceflight analog to investigate skeletal muscle alterations induced by unloading, to test countermeasures designed to combat unloading-induced changes, and to explore the molecular processes underpinning inactivity-induced muscle atrophy (Rullman et al., 2018; Fernandez-Gonzalo et al., 2020). Despite the numerous advantages of this model, performing bed rest studies of long duration is extremely challenging due to staff- and economic-related constraints. Only large national or international space agencies can afford to run long-duration bed rest campaigns, which include a rather limited number of subjects per intervention group. The small sample size is indeed a significant constraint when exploring individual variability in bed rest studies (Scott et al., 2021). Furthermore, the lack of a genuine control group in bed rest studies, i.e., an ambulatory group, has limited the validity of any past attempt to detect individual responses to bed rest interventions (Atkinson and Batterham, 2015; Scott et al., 2021). Overcoming these limitations could translate into improved health management of astronauts and optimized individual programs to counteract the negative effects of unloading both during space missions and on Earth (Scott et al., 2021).

A unique opportunity to address individual variability upon bed rest responses is the Planica bed rest program, where three studies lasting 10 or 21 days have been performed using identical pre- and post-bed rest tests under strictly-controlled conditions (Keramidas et al., 2016; McDonnell et al., 2019, 2020). In the three studies, each participant completed three interventions [i.e., hypoxic ambulation (HAMB), normoxic bed rest (NBR), and hypoxic bed rest (HBR)] in a randomized, cross-over design. Combining the three studies offers one of the largest reported bed rest datasets to date, increasing the statistical power to analyze the individual variability in skeletal muscle outcomes after bed rest.

In the current report, we combined the results from three bed rest studies performed at the Planica facility to evaluate changes in skeletal muscle mass and function induced by 10 and 21 days of bed rest with/without a hypoxic environment in a cohort of 35 participants. Then, we investigated the individual response in the variables showing robust changes to bed rest and assessed the potential moderators that may explain the variability across individuals. Given the current knowledge in bed rest-induced muscle alterations, we hypothesized that the loss of knee extension strength and reduction in muscle mass in both the thigh and the calf would be the muscle features most markedly affected by bed rest. We further hypothesized that changes in knee extension strength and muscle areas would show clinically relevant individual variability that is influenced by baseline levels and energy intake during the intervention.

## Materials and Methods

### General Study Design

Data were collected from three studies during the following periods: LunHab (from March to September 2011), PlanHab (from September 2012 to October 2013), and FemHab (from November 2013 to May 2014). These three bed rest studies were conducted at the Planica facility in the Olympic Sport Centre Planica, Ratece, Slovenia. In each study, subjects participated in three experimental campaigns in a counterbalanced randomized, cross-over design: NBR, HBR, and HAMB (the hypoxic conditions corresponded to an altitude of 4,000 m). The ambient conditions for each study are detailed in Supplementary Table 1. In both FemHab and LunHab each intervention lasted 10 days, while in the PlanHab study, the interventions had a duration of 21 days. The detailed study protocols have been described elsewhere (Debevec et al., 2014; McDonnell et al., 2019, 2020). In the current study, data related to muscle function, muscle area, body composition, and caloric intake from the three individual studies were merged into a single database, where subjects were grouped according to the particular intervention, irrespective of the study of origin. To be included in the final database, a participant had to complete at least two of the three campaigns. Then, data were analyzed for each variable as described below, and individual response was calculated. Finally, we analyzed potential moderators that could have influenced the subjects’ individual response.

### Participants

Inclusion and exclusion criteria for PlanHab, LunHab, and FemHab have been described in detail elsewhere (Debevec et al., 2014; McDonnell et al., 2019, 2020), and those criteria followed the European Space Agency (ESA) guidelines (Heer et al., 2009). Briefly, following a reply to a national advertisement, participants were provided with a detailed document outlining the specifics of the study. If they were interested and understood the protocol, they were then invited to a panel interview with at least 3 experienced researchers. From the initial pool of participants, a minimum of 20 were chosen in each study, and took part in a familiarization weekend at the Planica facility. This weekend provided an excellent opportunity to observe the participants interact, take part in experiments and deal with researchers. The final inclusion of the successful participants was based on the ESA guidelines, their initial interview and their performance during the familiarization. Once the participants were selected, they signed a written informed consent to participate in the particular study. The three studies conformed to the standards set by the Declaration of Helsinki. The procedures were approved by the Committee for Medical Ethics at the Ministry of Health (Republic of Slovenia; approval numbers: 205/2/11 and 88/04/12).

### Intervention Procedures

With the exception of the length of intervention (10-day interventions in the LunHab and FemHab studies; 21 day interventions in the PlanHab study) and participant sex (males in the LunHab and PlanHab studies, and females in the FemHab study), the protocol of the three interventions (HAMB, HBR, and NBR) was similar in all three studies. The studies were designed as cross-over repeated measures, such that each subject participated in all three interventions. Each study comprised three research campaigns, during which all subjects were confined to the Olympic Sport Centre. In the first campaign, the participants were randomly assigned to an intervention, and in the following two campaigns, they were then exposed to the remaining interventions. In any given research campaign (in all three studies), all three interventions were conducted simultaneously. The washout period between interventions was a minimum of 1-month for the 10-day interventions (LunHab and FemHab studies), and a minimum of 4 months for the 21-day interventions (PlanHab study). This ensured recovery of the participants taking part in more than one intervention (Convertino et al., 1985; Sandler et al., 1988). Briefly, during an ambulatory pre-intervention period of 5 (LunHab, FemHab) or 7 (PlanHab) days, subjects acclimated to the regime requested during the studies (i.e., sleep/wake cycle, nutrition, etc.), and baseline experimental measurements were obtained. This was followed by the intervention period, i.e., NBR, HBR, or HAMB. Finally, upon completion of the intervention, participants were requested to remain at the facility for an additional 4 to 7 days, so that post-intervention measurements could be obtained. For those who had completed a bed rest intervention, this also allowed safe re-ambulation. During the interventions, participants adhered to a strict daily schedule. They were awakened at 07:00 AM, with lights out at 11:00 PM. Napping during the day was not allowed, and participants in the HAMB interventions had to maintain a seated upright or standing position during the day (i.e., feet had to be in contact with the floor at all times). During the bed rest interventions (NBR and HBR), the participants had to maintain a strict horizontal bed rest. All daily activities were carried out in the horizontal position. The participants could use one pillow for head support, and were allowed to support themselves on an elbow during meals. Physical activity, apart from changing positions from supine to prone or lateral, was not permitted during the bed rest interventions. To ensure compliance to the bed rest protocol, participants were monitored at all times using continuous closed-circuit television, and by the medical staff. During the HAMB intervention the participants performed two daily low-intensity physical activity sessions to mimic their previous habitual daily activity. In LunHab, the participants performed 30 min of a stepping exercise in the morning (heart rate, HR: 115.9 ± 3.3) and afternoon (HR: 112 ± 2.7; McDonnell et al., 2014). The exercise mode was varied for PlanHab and FemHab to offer variety (stepping, cycling, or dancing), and it was rotated in the afternoon to avoid monotony. The participants always took part in a stepping exercise in the morning and chose their preferred activity for the afternoon. The average HR for both morning and afternoon sessions in PlanHab was 124 ± 9 (Keramidas et al., 2016). The FemHab participants HR response to the activity was 131 ± 10 (Debevec et al., 2016). The target heart rate for each of these exercise sessions was equivalent to that attained at 50% of the parpitants peak power out during a hypoxic (4,000 m) cycle ergormetry test to exhaustion.

### Oxygen Depleted Gas

During the HBR and HAMB interventions, normobaric hypoxia was maintained with a vacuum pressure swing adsorption system (VPSA: b-Cat, Tiel, Netherlands), which delivered oxygen-depleted gas to the hypoxic area of the Planica facility. The oxygen content of each room in the facility was assessed by the VPSA system at 15 min intervals throughout the interventions. If the concentration measured in the rooms was above the target fraction of ambient O_2_ (F_I_O_2_: 0.142) a hypoxic gas mixture generated by the VPSA system was pumped to the desired room. However, if the ambient O_2_ was below the target, the delivery of external normoxic air was iniated to that area. As a safety precaution, the participants also carried a personal O_2_ analyzer (PGM-1100; Rae Systems, San Jose, CA, United States), which would provide immediate feedback of the ambient F_I_O_2_.

### Muscle Function

The Biodex S4 Pro isometric dynamometer (Biodex Medical Systems, System Pro 4, Shirley, New York, United States) was used to assess muscle function through a maximal isometric voluntary contraction before and after each campaign. The following joints and angles of assessment were measured unilaterally: ankle: 15° plantar flexion; knee: 60° and the elbow 60°. The dynamometer was calibrated prior to any testing. The joint center of rotation was aligned with the axis of rotation of the dynamometer and the participants were requested to conduct a maximal-effort muscle action. The protocol was standardized according to the ESA standard operating procedures and consisted of a 5-s isometric contraction of the agonist followed by a 5-s recovery, then a 5-s isometric contraction of the antagonist followed by a 5-s recovery. This pattern was repeated until 5 contractions of each muscle group were completed. The maximal torque was described as the peak isometric force attained during 50 ms epochs in any of the five 5-s contractions.

### Muscle and Fat Area

The muscle cross sectional area (CSA) and fat area in the thigh and calf were assessed using peripheral quantitative computer tomography (pQCT, XCT3000 Stratec Medizintechnik, Pforzheim, Germany). The participants’ non-dominant leg was fully extended and positioned within the device while they lay supine on an adjacent medical bed. The foot and thigh were fixed to the supporting structures of the pQCT device. Muscle CSA and fat area were assessed before the start of each intervention and on the final day (day 10 in LunHab and FemHab; day 21 in PlanHab). pQCT scans were obtained at 66% of the tibial length (from the ankle) and at 33% of the femoral length (from the knee) and the resultant images were analyzed with the manufacturers’ software (XCT3000 version 5.4) and data stored for subsequent analysis.

### Body Composition

Whole body and regional fat and lean mass were assessed with dual-energy X-ray absorptiometry (DEXA) before and immediately after each campaign using a Hologic fan-beam densitometer (Discovery W-QDR series, Hologic, Bedford, United States). DEXA scans were analyzed with Hologic DOS software (Hologic APEX System Software, version 3.1.2). During the DEXA scans, participants were dressed in minimal clothing (i.e., *t*-shirt and underwear). Daily calibration was conducted with the soft tissue calibration modules provided by the manufacturer. Participants were scanned supine in a fasted state and well rested. The same researcher analyzed all scans. The regions of interest used for the analysis were: (a) upper arm (elbow joint center–gleno-humeral joint center; lower–upper boundary); (b) thigh (knee joint center–acetabulo-femoral joint center); and (c) lower leg: (ankle joint center–knee joint center).

### Diet and Energy Intake

A strictly controlled, standardized diet was adapted to the individual participant’s requirements to facilitate energy balance during the study (Debevec et al., 2014). Daily resting energy expenditure was estimated for each participant using the modified Harris–Benedict equation (Roza and Shizgal, 1984) and multiplied by a physical activity level factor (PAL) to calculate daily dietary energy requirement according to the study phase. During the ambulatory phases (Pre and Post intervention and HAMB confinement) a PAL of 1.4 was used, with a PAL of 1.2 applied for the bed rest phases (NBR and HBR confinement), according to standard guidelines (Sundblad and Orlov, 2014). The target macronutrient composition of the diet, expressed as a proportion of total dietary energy intake, was 55% carbohydrate, 30% fat, and 15% protein. Additionally, the diet aimed to provide 1.2 g of protein/kg body weight and a sodium intake of <3,500 mg per day, and was supplemented with vitamin D3 (1,000 IU/day).

The standard menu was devised using a web-based application “Open Platform for Clinical Nutrition” (www.opkp.si, Jozef Stefan Institute, Ljubljana, Slovenia), and repeated during each subsequent campaign to ensure the consumption of identical meals on the same days of each respective campaign. Five meals (breakfast, morning snack, lunch, afternoon snack, and dinner) were served daily and at the same time of the day throughout the campaigns. Meals were based on the standard Slovene diet and participants were encouraged to eat all of the food provided. Moreover, a daily intake of >28.5 ml of fluid per kg body weight was encouraged through *ad libitum* consumption of water and unsweetened fruit tea. No additional food or drink (outside of the provided menu) were allowed, including consumption of alcohol or caffeine-containing beverages, but participants could choose to consume less food than was provided. During plating of the meals, each food item was weighed on a precision (±0.1 g) scale (TPT 6C, Libela ELSI, Celje, Slovenia) connected to a custom-developed, computer-based food recording and analysis system (Piki 2.0, Faculty of Computer science, University of Ljubljana, Ljubljana, Slovenia), with unconsumed food items being re-weighed and the value deducted from the initial weight to provide actual food intake.

The daily difference between actual and targeted energy intake was calculated for each participant and averaged across each intervention. In addition, the coefficient of variation (CV; standard deviation divided by the mean of the daily differences in the actual vs. targeted energy intake) was calculated for each participant and intervention.

### Data Analysis

Potential differences in pre-to-post for all outcome measures in the three campaigns (HAMB, NBR, and HBR), were analyzed parametrically in a pairwise fashion and standardized effect sizes were calculated, where values of <0.1, 0.1–0.3, 0.3–0.5, and >0.5 were deemed as trivial, small, moderate, and large, respectively, (Hopkins, 2006). In addition, a mixed-effects model with study (i.e., PlanHab, FemHab, and LunHab) and intervention (i.e., HAMB, NBR, and HBR) as fixed-factors and subject as a random factor, was performed using the change scores (i.e., difference post – pre) of all available variables. Significant interactions were followed by Tukey *post-hoc* tests to correct for multiple comparisons. Individual variability was investigated by using the definition suggested by Hopkins (2015) and Atkinson and Batterham (2015), that is; SD_IR_ = √(SD${}_{\text{Exp}}^{2}$ – SD${}_{\text{Con}}^{2}$), where SD_IR_ is the standard deviation for the individual response and SD_Exp_ and SD_Con_ are the standard deviations of the change score in the experimental (NBR or HBR) and the control (HAMB) groups, respectively. In the event of a greater SD_Con_ vs. SD_Exp_, and given that it is not possible to calculate the square root of a negative number, the sign was changed to perform the square root but the final result was considered negative (Hopkins, 2015). The typical overall effect of NBR and HBR on an individual was calculated as the mean intervention effect (vs. HAMB) ± SD_IR_. Pearson’s correlation coefficient (r) was used to investigate the repeatability of responses across campaigns (i.e., during NBR and HBR) and to follow up any variable showing individual response. These statistical analyses were performed using Prism 7 for Mac OS X (GraphPad Software Inc, San Diego, CA, United States). Principal component analysis (PCA) and Orthogonal Partial Least Squares (OPLS) regression on normalized change scores were performed on R version 3.5.3 – “Great Truth,” using the factominer and rOPLS libraries (Thévenot et al., 2015). Data series from participants with >75% valid outcome measures across the campaigns were used. For the OPLS analysis, singular missing values were imputed using a weighted average of the 5 nearest neighbors (k-nearest neighbors algorithm; kNN; Torgo, 2016), which was used for only two data points. Confidence intervals and *p*-values were generated numerically through boot-strapped cross-validation. The level of significance was set at 5% (*P* < 0.05). Data are presented as means ± standard deviation (SD) or as relative changes in % units.

## Results

After filtering for inclusion criteria (i.e., at least two interventions performed within one study), the number of participants included in the database was 35, with 9 in FemHab, 12 in LunHab, and 14 in PlanHab. Participants’ baseline characteristics are displayed in Table 1. The combination of available PlanHab, LunHab and FemHab body composition and skeletal muscle data translated into a database with 19 variables with results from the three studies, with the exception of fat area in thigh and calf during PlanHab (Supplementary Table 2). In addition, information regarding nutritional status (i.e., change score and CV of actual vs. targeted energy intake; Supplementary Table 2) was collected for the three studies. Results for some of these outcomes from each individual study have been published elsewhere (Debevec et al., 2014; McDonnell et al., 2019, 2020; Mekjavic et al., 2020).

**TABLE 1:** Descriptive characteristics of the pariticpants included in the analysis.

	*n*	Age (year)	Height (cm)	Weight (kg)
PlanHab	14	26.4 ± 5.2	179.6 ± 5.1	76.9 ± 10.8
FemHab	9	26.7 ± 3.7	167.5 ± 6.1	60.0 ± 7.3
LunHab	12	24.1 ± 2.2	180.2 ± 6.7	72.4 ± 11.6
All combined	35	25.7 ± 4.1	176.7 ± 8.0	71.0 ± 12.1

*PlanHab, Planetary Habitat Simulation; FemHab, Female Habitat Simulation; and LunHab, Lunar Habitat Simulation.*

### Changes Induced by the Three Interventions

The first aim of this analysis was to establish combined effect sizes in each of the three interventions performed. Thus, we ran a comparison of pre vs. post values for HAMB, NBR, and HBR independently of the study of origin (Supplementary Table 3). Many of the strength-related measurements showed a significant pre-to-post decrease after bed rest. The magnitude of the standardized effect sizes (ES) was small in most cases, except for knee extension (KE) torque (NBR; −8.8%, large ES, HBR; −7.0%, moderate ES), knee flexor torque (NBR; −6.2%, HBR; −7.1%, both moderate ES), and dorsiflexor (HBR; −10.5%, moderate ES; Supplementary Table 3). The results also indicated significant muscle area decrements for thigh and calf muscles after the three interventions, although the effect sizes for HAMB were small (change lower than −2.9%), while the effect sizes for NBR and HBR for the same outcomes were between 2 and 4 times greater (change between −4.7 and −10.3%; Supplementary Table 3). Several body composition outcomes were significantly reduced after the three interventions with effect sizes of low magnitude (trivial-small), and comparable across HAMB, NBR, and HBR (Supplementary Table 3).

**FIGURE 1:**
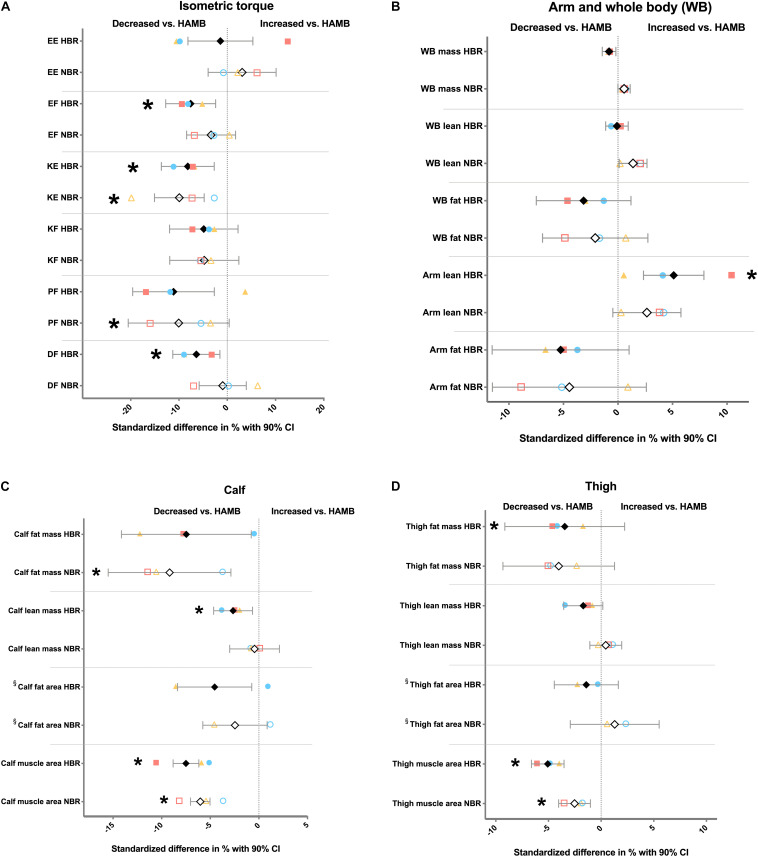
Differences in change scores expressed as relative values (i.e., standardized difference) between hypoxic ambulation (HAMB) and normoxic or hypoxic bed rest (NBR and HBR, respectively) for **(A)** force variables; **(B)** arm and whole-body (WB) variables; **(C)** calf-related variables; and **(D)** thigh-related variables. Open symbols; % difference between NBR vs. HAMB. Full symbols; % difference between HBR vs. HAMB. Black diamonds, all subjects from FemHab, LunHab and PlanHab combined. Blue circles; FemHab. Yellow triangles; LunHab. Red squares; PlanHab. CI, confidence interval; EE, elbow extension; EF, elbow flexion; KE, knee extension; KF, knee flexion; PF, plantar flexion; and DF, dorsiflexion. * denotes significant difference (*P* < 0.05) in change score in absolute values vs. HAMB. ^§^ no PlanHab data available for this variable.

Thereafter, we sought to identify variables significantly and consistently affected by the two bed rest campaigns (i.e., NBR and HBR) in relation to HAMB. Although the HAMB group may not be seen as a regular control group due to the hypoxic condition, it allowed us to compare the data controlling not only for genetic factors (each subject was his/her own control), but also for intervention time and energy intake, traits that can be important for the outcomes under study. It should be acknowledged that by employing HAMB as the control group we used a rather conservative approach, since the majority of the detected changes in HAMB went in the same direction (i.e., reduction) as the changes during the bed rest campaigns (Supplementary Table 3), and therefore may have masked some of the effects of bed rest. Keeping this in consideration, our investigation indicated that the change-scores significantly differed in several muscle and body composition outcomes (Figures 1A–D). However, there were only three variables where the difference between HAMB vs. NBR/HBR was true for both bed rest interventions, i.e., knee extension torque (KE; *P* < 0.02), thigh muscle area (*P* < 0.014), and calf muscle area (*P* < 0.0001), indicating reduced force and muscle mass with unloading.

Since FemHab, LunHab, and PlanHab differed in terms of campaign length and participant sex, we further analyzed the effect of study on the three outcomes highlighted by our previous analysis, i.e., KE torque, and thigh and calf muscle area. To this end, we conducted a mixed-effects model (see section “Materials and Methods”). KE torque decrements were larger in men (i.e., PlanHab and LunHab) than women (FemHab; main effect of study, *P* = 0.0019; *F* = 6.7). Also, both bed rest interventions (i.e., NBR and HBR) triggered greater losses on KE torque than HAMB (main effect of intervention, *P* = 0.0097; *F* = 4.9). Regarding thigh muscle area, longer bed rest periods exacerbated muscle loss, as indicated by a main effect of study (*P* < 0.0001; *F* = 14.6), with PlanHab subjects suffering an overall greater thigh muscle atrophy than LunHab and FemHab participants. Both bed rest campaigns induced greater overall thigh muscle atrophy than HAMB (main effect of intervention, *P* = 0.0001; *F* = 12.9). Calf muscle area results showed a significant interaction study × intervention (*P* < 0.0001; *F* = 9.8) where *post-hoc* analyses revealed that the longer (21 days) PlanHab bed rest interventions induced greater calf muscle loss than those in LunHab and FemHab (10 days; *P* < 0.0001). In addition, the magnitude of calf muscle loss during HAMB was significantly lower than NBR and HBR in LunHab and PlanHab (*P* < 0.008), and tended to be lower than HBR in FemHab (*P* = 0.057). The effect of NBR and HBR was not significantly different in any study. The small effect sizes of HAMB were very similar across studies.

### Individual Variability

As hypothesized, our analyses demonstrated that muscle mass and force are the variables more heavily affected by bed rest. Consequently, we investigated individual variability in the response to bed rest in KE torque, and thigh and calf muscle area by calculating the individual response by means of SD_IR_ (see section “Materials and Methods”). The SD_IR_ for KE torque was 18.2 and 22.1 for NBR and HBR, respectively. This translates into a typical overall effect ranging from −2.0 to −38.3 Nm (−0.9 to −17.8%) for NBR, and from 6.0 to −38.2 Nm (2.8 to −17.9%) for HBR. For calf muscle area, the SD_IR_ was 344.6 and 332.1 for NBR and HBR, respectively, which would indicate that the typical overall effect ranges from −142.8 to −831.9 mm^2^ (−1.9 to −11.3%) for NBR, and from −238.2 to −902.3 mm^2^ (−3.3 to −12.4%) for HBR. SD_IR_ values for thigh muscle area were −147.9 for NBR (note the negative value of SD_IR_) and 371.2 for HBR, which translates into a typical overall effect ranging from −150.1 to −445.8 mm^2^ (−1.4 to −4.2%) for NBR, and from −211.9 and −954.2 mm^2^ (−2.0 to −8.9%) for HBR. The SD_IR_ results for all variables included in this study are shown in Supplementary Table 4. Overall, and considering previous reports (Fearon et al., 2011), these results indicate that the individual response for KE torque and calf muscle area are indeed clinically relevant (i.e., range of the typical overall effect greater than 5%), while the magnitude of individual response expected for thigh muscle area is not assessed as being as clinically important, especially after NBR.

### Repeatability

**FIGURE 2:**
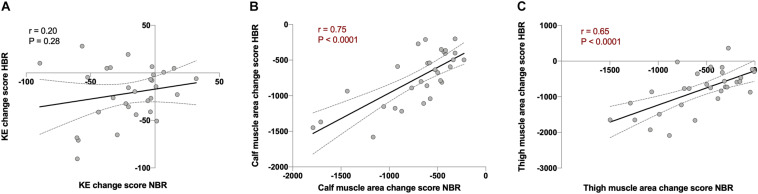
Repeatability analysis using Pearson’s correlations between the change score of normoxic bed rest (NBR) vs. hypoxic bed rest (HBR) in **(A)** knee extension torque (KE; in Nm, *r* = 0.20, and *P* = 0.28), **(B)** calf muscle area (in mm^2^, *r* = 0.75, and *P* < 0.0001), and **(C)** thigh muscle area (in mm^2^, *r* = 0.65, and *P* < 0.0001) in FemHab, LunHab, and PlanHab bed rest studies combined. Dotted lines represent 95% confident intervals.

**FIGURE 3:**
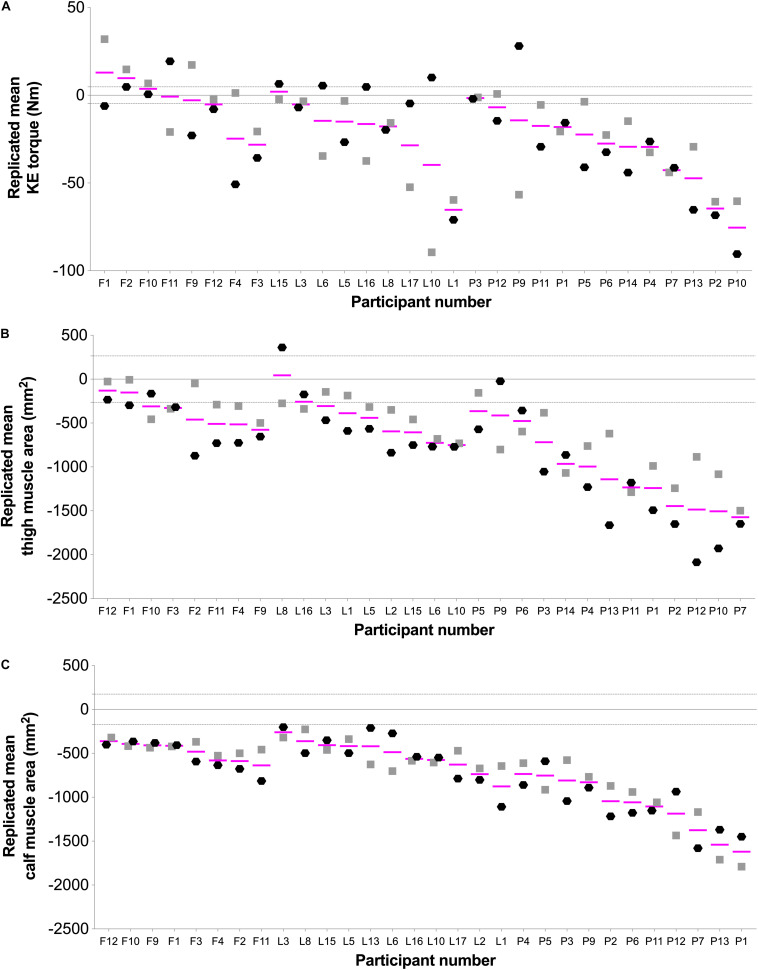
Individual changes showing the repeatability response in **(A)** knee extension (KE) torque, **(B)** thigh muscle area, and **(C)** calf muscle area. Black hexagons (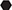; hypoxic) and gray squares (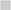; normoxic) indicate change scores (post minus pre) for the responses to bed rest interventions. Pink lines (—) represent each participant’s replicated mean. Gray-dashed lines represent the standardized minimally clinically important difference, which was calculated by multiplying 0.1 by the baseline between-subject standard deviation (Atkinson and Batterham, 2015; Goltz et al., 2018, 2019). Letters in X axis indicate FemHab (F), LunHab (L), or PlanHab (P) study subjects, and are ordered from lower to higher average loss after bed rest within each study for each particular variable.

Once an individual response was identified, we investigated the repeatability of such a response by analyzing the magnitude of alterations each participant underwent during both bed rest interventions. The individual response during NBR and HBR (i.e., repeatability) correlated highly to each other for calf (*r* = 0.75, *P* < 0.0001) and thigh (*r* = 0.65, *P* < 0.0001) muscle area, but not for KE torque (Figures 2, 3). These data suggest that the magnitude of muscle atrophy for a particular individual after a bed rest intervention could be predicted quite accurately by the muscle mass lost in a previous unloading intervention. However, this type of prediction would not be possible for maximal torque production.

### Moderators of the Individual Variability

**FIGURE 4:**
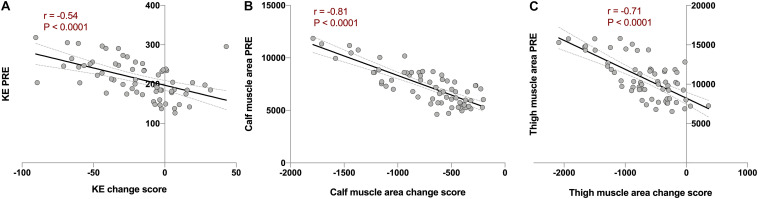
Analysis of baseline value as a moderator of individual variability using Pearson’s correlations between combined change scores in normoxic (NBR) and hypoxic (HBR) bed rest campaigns vs. PRE values before the corresponding intervention in **(A)** knee extension torque (KE; in Nm. *r* = −0.54, *P* < 0.0001), **(B)** calf muscle area (in mm^2^, *r* = −0.81, *P* < 0.0001), and **(C)** thigh muscle area (in mm^2^, *r* = −0.71, *P* < 0.0001) in FemHab, LunHab, and PlanHab bed rest studies combined. Dotted lines represent 95% confident intervals.

The next step we took was to analyze potential factors, or moderators, that could influence the individual response. Our first candidate was baseline values. As hypothesized, the change score in KE torque, and thigh and calf muscle area showed strong, negative and significant correlations with baseline values (*r* between−0.5 and −0.9; *P* < 0.001). This was true for both NBR and HBR combined (Figures 4A–C) and when analyzed separately (Supplementary Figure 1). When the analyses were performed considering the relative change (% from PRE) instead of the change score, the significant correlations remained (*r* between 0.45 and 0.47; *P* < 0.0005). This clearly points out that the stronger and/or bigger (in terms of muscle mass) an individual is, the more force and muscle mass would be lost under microgravity conditions.

The next factor analyzed was dietary energy intake, investigated as actual intake in relation to targeted intake in terms of both change score and CV across days. No diet variable correlated with the change scores of thigh muscle area or KE torque, and only the deviations in energy intake in relative terms showed a weak significant correlation with the relative loss of calf muscle area decrements (*r* = −0.258; *P* = 0.048). The change score of targeted vs. actual energy intake correlated in a positive fashion with whole-body (WB) mass in both relative (*r* = 0.322; *P* = 0.013) and absolute values (*r* = 0.317; *P* = 0.014). Deviation in energy intake in relative values also correlated with whole body mass (*r* = 0.339; *P* = 0.008, WB mass in absolute values, and *r* = 0.349; *P* = 0.006, WB mass in relative values). The relative change in whole-body fat mass after the bed rest interventions correlated with the absolute (*r* = 0.316; *P* = 0.015) and relative (*r* = 0.309; *P* = 0.016) deviations in energy intake. Taken together, these results indicate that while deviations from the targeted energy intake seem to have a role in the degree of weight change and fat accumulation, their role in muscle atrophy and loss of force during bed rest is very limited.

**FIGURE 5:**
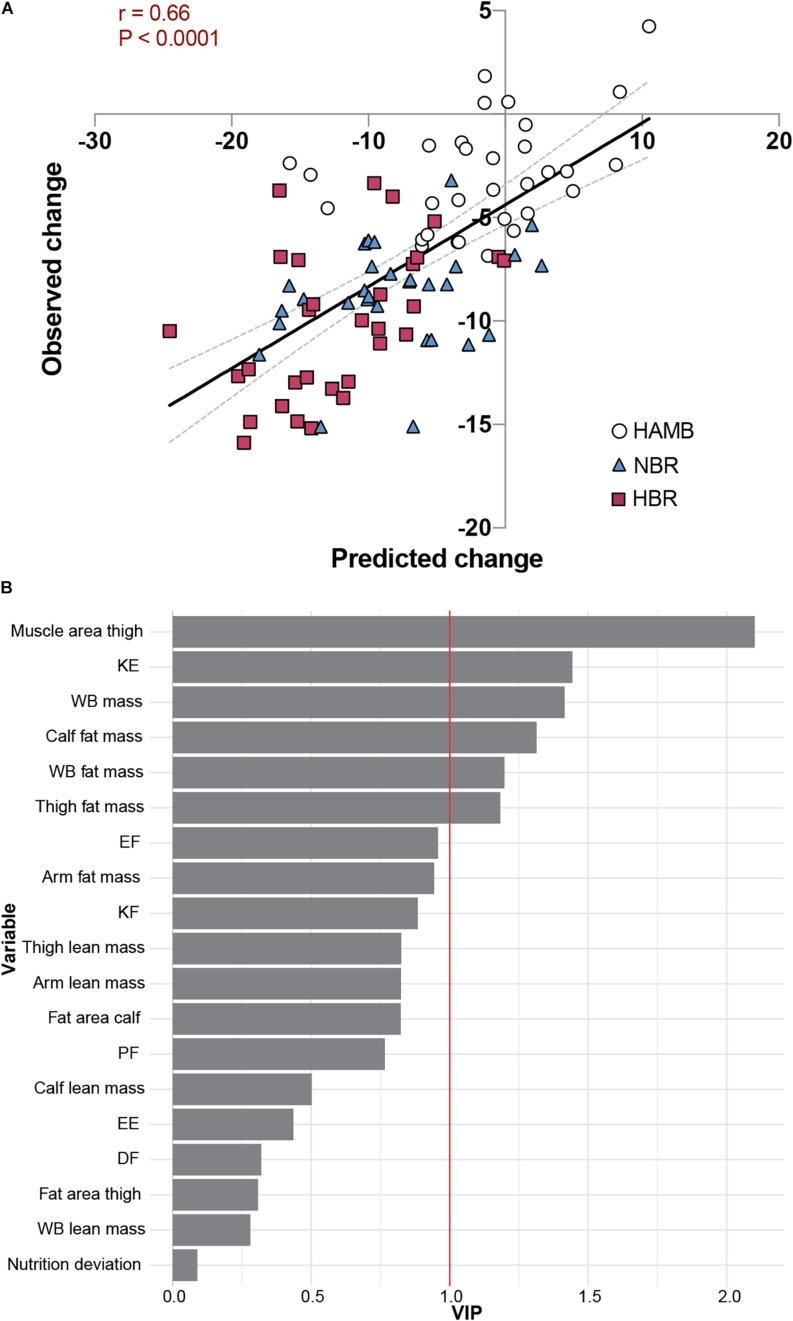
Global, exploratory approach to the changes in calf muscle area in Planica bed rest studies. HAMB; hypoxic ambulation, HBR; hypoxic bed rest, and NBR; normoxic bed rest. **(A)** Scatter plot of predicted and observed changes in calf muscle area using our OPLS regression (*r* = 0.66, *P* < 0.001). Dotted lines represent 95% confident intervals. **(B)** Variable Importance in Projection (VIP) showing the rank of contributing variables to the successful modelling (KE, knee extension torque, WB; whole body, EF; elbow flexion torque, KF; knee flexion torque, PF; plantar flexion torque, EE; elbow extension torque, and DF; dorsiflexion torque).

Finally, we employed a global, exploratory approach to assess the changes in calf muscle area in the Planica bed rest studies. This was based on our observation indicating that calf muscle area is the most consistently altered outcome measure across the studies and bed rest campaigns, and also one of the outcomes with the most significant inter-individual variability. To this end, we first analyzed the degree of covariance amongst the change scores across the campaigns and studies using PCA (Supplementary Figure 2A). This revealed a modest degree of covariance in the change scores with 21.9 and 16.6% captured by the first two principal components and with little variable clustering. In line with this, calf muscle area contributed equally to these components (coefficient of regression 0.47 and 0.40, respectively). Next, we utilized the supervised machine learning method OPLS regression to explore if the global change scores could be used to model the change in calf muscle area. Based on calf area being equally correlated to both PC1 and PC2 we chose to retain all variables from the PCA to test in the OPLS-model. After 10,000-fold cross validation, a successful OPLS regression (*P* < 0.01) with 1 predictive and 1 orthogonal component with a predicted *r*^2^-value of 0.43 for calf muscle area was generated (Figure 5A and Supplementary Figure 2B). Variables contributing most to the successful modeling were “Thigh muscle area,” “KE torque,” and “whole-body mass,” based on Variable Importance in Projection (Figure 5B). This was corroborated by direct univariate correlation analysis between change scores of calf muscle area vs. thigh muscle area, KE torque and whole-body mass, rendering correlation coefficients of 0.593, 0.285, and 0.262, respectively. Comparable correlation coefficients were obtained when the different campaigns were analyzed separately.

## Discussion

By combining individual data from three studies, we generated the largest muscle and body composition bed rest dataset to date. Of the 19 variables examined, KE isometric torque, and calf and thigh muscle area were affected by bed rest the most. The main finding of this study is that there was clinically relevant individual variability in KE torque, and calf and thigh muscle area, and this individual response was repeatable across bed rest interventions, at least for muscle mass readouts. The results also showed that baseline values, but not deviations in the tailored energy intake, seemed to be a moderator of the variability. Another moderator of the individual response to calf muscle area loss with bed rest, was a global bed rest campaign response including all of the other variables studied, where muscle mass and function changes contributed the most.

The current analysis translated into approximately a 3-times greater number of observations compared to what is commonly found in bed rest studies (Alkner and Tesch, 2004; Trappe et al., 2007a; Blottner et al., 2020). The analyses performed indicated that the mass of the muscles involved in posture and locomotion (i.e., thigh and calf) and the function of the knee extensor muscles are the outcomes more robustly affected by bed rest, supporting previous results (Pavy-Le Traon et al., 2007). However, other outcome measures that have been used to assess the effects of bed rest on skeletal muscle showed effect sizes of small magnitude and substantially lower reproducibility both across trials and within individual subjects [e.g., lean whole-body/leg mass (Pavy-Le Traon et al., 2007; English et al., 2016)]. This indicates that these variables are more influenced by the study design, method limitations, or other external factors, rather than by unloading *per se*, and therefore should be used and interpreted with caution when examining the consequences of bed rest in skeletal muscle.

Anecdotal evidence has suggested that there is substantial individual variability in physiological variables following bed rest, including losses in muscle mass and function (Akima et al., 1997; Downs et al., 2016; Scott et al., 2021). However, the limited number of participants included in previous studies and the lack of an ambulatory control group prevented any firm conclusion. In contrast, the current study provided a framework including a control group and around 30 observations per variable, which together offered a unique opportunity to study individual variability in response to bed rest. This strategy allowed us to control for heritable factors, intervention time and energy intake, and to use a conservative control-arm for each subject in the form of HAMB under the same experimental set-up/environment as the bed rest interventions (see “Results” section for details). Thus, a methodology described by experts in the field (Atkinson and Batterham, 2015; Hopkins, 2015), and used in other physiological settings to investigate individual variability to an intervention (Goltz et al., 2018, 2019), was employed for the three variables showing more robust changes to bed rest. The data showed that if a random individual would undergo bed rest in similar conditions as the ones used in the Planica studies, a loss ranging from 0 to 17% and from 2 to 12% could be expected for KE torque and calf muscle area, respectively, which could be interpreted as clinically relevant (Fearon et al., 2011). When it comes to thigh muscle area, the negative SD_IR_ for NBR indicated that there was more variability in response to HAMB than NBR; yet, the individual response in thigh muscle area losses after HBR would be close to a clinically relevant threshold. A factor to consider is that during bed rest, there is a complete standardization (i.e., absence) of the mechanical load, while in HAMB, despite potential drops in physical activity induced by the confinement, and thus mechanical load, there was a retained behavioral variance, i.e., some participants would move around more than others, augmenting the mechanical load variability across individuals.

The differences in the standardization of the mechanical load during NBR vs. HAMB could partly explain the negative SD_IR_ noted following NBR, since a greater regression to the mean phenomenon could be induced by NBR than by HAMB. Yet, if this phenomenon was completely true, it would have appeared for the HBR intervention as well. An explanation for this could be the differences in the magnitude of the effect size alterations for thigh muscle area during NBR and HBR, with higher values (almost double) for HBR. Altogether, these results highlight that individual variability estimations are mainly relevant in situations where the effect size of the intervention arm is substantially larger than in the control group, as well as the undisputable necessity to include a control arm in the experiments (i.e., ambulatory group in bed rest studies).

Repeatability was investigated by comparing the change scores in KE torque, and calf and thigh muscle area in NBR vs. HBR. Despite the trivial/small impact of hypoxia in bed rest-induced alterations, the current strategy to investigate repeatability allowed us to address whether the individual variability to a bed rest intervention was mainly explained by inter-campaign/tests effects, i.e., external, random factors (i.e., different individual response in NBR vs. HBR), or if the variability could be explained by intra-subject factors, and therefore could be considered as real individual variability (i.e., similar individual response in NBR and HBR). We report for the first time a high degree of repeatability for calf and thigh muscle area change scores, but not for KE torque, after bed rest. Therefore, while the individual variability reported above for bed rest-induced muscle atrophy can be considered as real intrinsic intra-individual variation in response to microgravity, other random/extrinsic elements (e.g., learning effects) seemed to have affected the losses in KE torque. Apart from any potential inter-test effect, the mismatch in repeatability between muscle mass and force losses could be partly explained by periodic variations in maximal force production (Ahtiainen et al., 2016). Such variations may be influenced by day-to-day differences in neuromuscular performance, psychological confounders or, to a lower degree in the current study design, variations in daily physical activity (Ahtiainen et al., 2016).

Once clinically-relevant individual variability was identified in specific outcomes, and the reproducibility confirmed across bed rest studies, factors that could influence the individual response were investigated, i.e., potential moderators of the individual response (Atkinson and Batterham, 2015). Given the available data, baseline values and deviations in energy intake from the targeted diet were examined. A strong, negative correlation between baseline values and loss of muscle force, and calf and thigh muscle area was found, suggesting that initial levels of force and muscle mass are important to explain the individual variability after bed rest. Although such relationships might be seen as a “regression to the mean” phenomenon (Bland and Altman, 1994; Linden, 2013), the physiological relevance of the current results should not be overlooked, more so when the correlations were still present after controlling for baseline levels. Indeed, the fact that the correlation was not found in the HAMB group does not support the proposal of a potential regression to the mean effect caused by sampling or methodological reasons. To interpret these data in relation to spaceflight, researchers should consider that while bigger and stronger individuals could lose more muscle mass and force during space missions, they would still have bigger safety margins to overcome the consequences of those space-induced alterations, as inferred in the past (Winnard et al., 2019).

The second potential moderator investigated was the deviation in energy intake from the targeted, individualized diet. The results showed that, in the context of a strictly controlled diet, there was practically no relationship between energy-intake deviations and muscle outcomes. Yet, deviations in the diet correlated with bed rest-induced changes in whole-body mass and whole-body fat mass. This is not surprising given that fat mass and body weight are heavily dependent on the overall energy intake, while muscle mass is regulated by protein intake and contractile activity (Longland et al., 2016). In the context of microgravity, muscle contractile activity seems to be the critical factor governing muscle mass during bed rest (Trappe et al., 2007b). The current data highlight that the results presented herein were not a consequence of any methodological artifact in diet registration or body composition and muscle mass/function testing, but of real biological origin.

The classic technique to analyze moderators of individual variability has been, as explained above for baseline values and diet deviations, to investigate the influence of one single factor on a particular outcome. However, given the current development of biostatistical and data-integration approaches, it could be more useful to investigate the overall signature of an intervention on the individual variability of a selected variable. For this approach to be valid, the intervention should be conducted under extremely controlled conditions, such that most of the environmental factors are accounted for. Thus, bed rest studies offer a unique opportunity to test and develop such moderator models. With this in mind, an exploratory approach to the changes in calf muscle area in the Planica bed rest studies was carried out to test the idea that the summatory effects of changes during bed rest (independently of their magnitude) in a considerable number of variables, could explain the change in calf muscle area. The calf muscle area was chosen for this set of analyses because (i) it is the most consistently altered outcome measure across the studies and bed rest interventions, (ii) it is one of the outcomes with the most significant inter-individual variability, and (iii) it presents a high degree of repeatability across bed rest interventions. In addition to the observations from the current experiments, calf muscle mass is one of the most investigated outcomes in the context of bed rest (Alkner and Tesch, 2004; Trappe et al., 2007b; Salanova et al., 2015; Blottner et al., 2020). Our analyses indicated that the variability in calf muscle area changes induced by bed rest could be moderately explained by the summatory effects of all of the other variables included in the database, with “thigh muscle area,” “KE torque,” and “whole-body mass” as the three top-ranked variables in the model. These results indicate that, despite minor or residual moderator role of the other variables when analyzed one-by-one, when they were merged, they accounted for ∼43% of the variance in calf muscle area changes induced by bed rest. Thus, the interindividual variance to bed rest is to an extent a global event where interindividual traits are shared across different physiological outcomes.

To fully interpret the results of the present study, there are some considerations that need to be taken into account. While we had access to individual traits for each individual participant, the analyses performed pooled studies with different bed rest duration (10 and 21 days) and sex. Although this could be seen as a limitation, the database introduced in this study is one of the biggest aggregated datasets, i.e., greatest number of observations on bed rest to date. Another factor to consider is the selection of HAMB as the control group. We acknowledge that HAMB is not the classic control group due to the soft intervention with hypoxia. Yet, the changes in most variables went in the same direction as those after bed rest, suggesting that this was a rather conservative approach, decreasing the risk of false-positive results to negligible levels. If anything, the current strategy might have masked some of the effects of bed rest. Despite these issues, this is the only set of bed rest studies using an ambulatory group for comparisons, which is paramount when investigating individual variability to an intervention (Atkinson and Batterham, 2015).

## Conclusion

Using the three studies performed at the Planica bed rest facility, clinically relevant individual variability was identified in changes in muscle force and mass. This individual variability was repeatable across bed rest interventions, at least for muscle mass alterations, and partly dependent on baseline values. In addition, the summatory effects of all of the variables analyzed became a fairly strong moderator of the variance in the calf muscle area changes after bed rest. The current results indicate clinically relevant individual variability in muscle responses to unloading/inactivity in the Planica bed rest campaigns. These data may serve as one of the cornerstones to develop (bio)markers of the individual response, which would offer new tools to improve health management of astronauts and to optimize individual programs to counteract the negative effects of unloading both during space missions and here on Earth.

## Data Availability Statement

The raw data supporting the conclusions of this article will be made available by the authors upon reasonable request.

## Ethics Statement

The studies involving human participants were reviewed and approved by Committee for Medical Ethics at the Ministry of Health (Republic of Slovenia). The patients/participants provided their written informed consent to participate in this study.

## Author Contributions

RF-G, AM, ER, and IBM conceived and designed the work. AM, IBM, ES, and IAM acquired the data. RF-G and ER analyzed and interpreted the data of the work and drafted the manuscript. RF-G, AM, ES, IAM, ER, and IBM critically revised the manuscript for important intellectual content. All authors contributed to the article and approved the submitted version.

## Conflict of Interest

The authors declare that the research was conducted in the absence of any commercial or financial relationships that could be construed as a potential conflict of interest.
