# Supplementary material for: Substantial and Reproducible Individual Variability in Skeletal Muscle Outcomes in the Cross-Over Designed Planica Bed Rest Program
Source: Front Physiol. 2021 Jul 16;12:676501. doi: 10.3389/fphys.2021.676501 (PMC8322684; doi:10.3389/fphys.2021.676501)
Supplement: Supplementary file 1 [file Image_1.pdf]

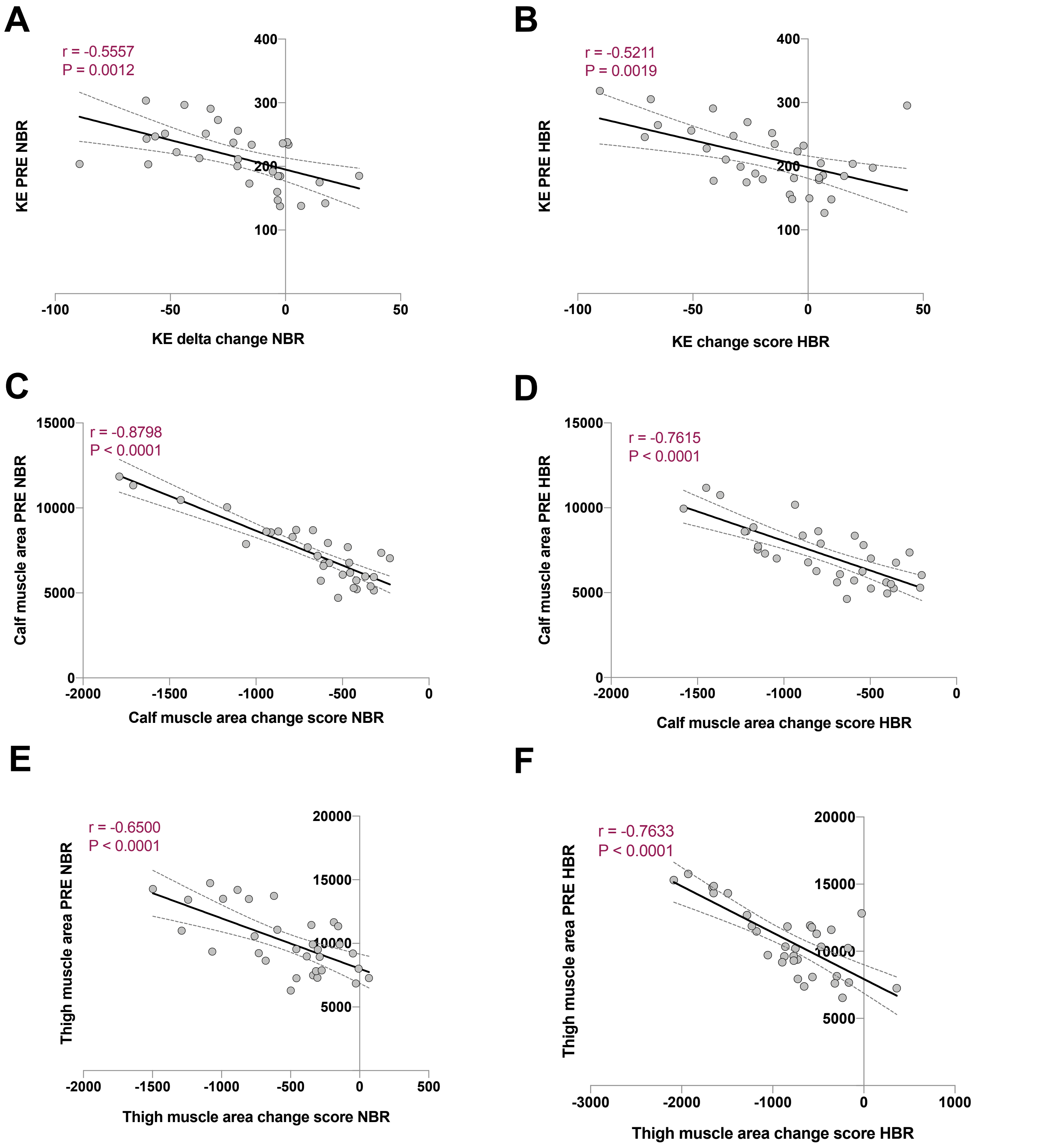

Supplemental Figure S1 – Pearson's correlations between the change score after normoxic (NBR) or hypoxic (HBR) bed rest vs. PRE values before the corresponding intervention in knee extension torque (KE; in Nm; A; NBR, B; HBR), calf muscle area (in mm^2; C; NBR, D; HBR), and thigh muscle area (in mm^2; E; NBR, F; HBR) in FemHab, LunHab and PlanHab bed rest studies combined. Dotted lines represent 95% confident intervals.
