# Supplementary figures and images for: Substantial and Reproducible Individual Variability in Skeletal Muscle Outcomes in the Cross-Over Designed Planica Bed Rest Program
Source: Front Physiol. 2021 Jul 16;12:676501. doi: 10.3389/fphys.2021.676501 (PMC8322684; doi:10.3389/fphys.2021.676501)

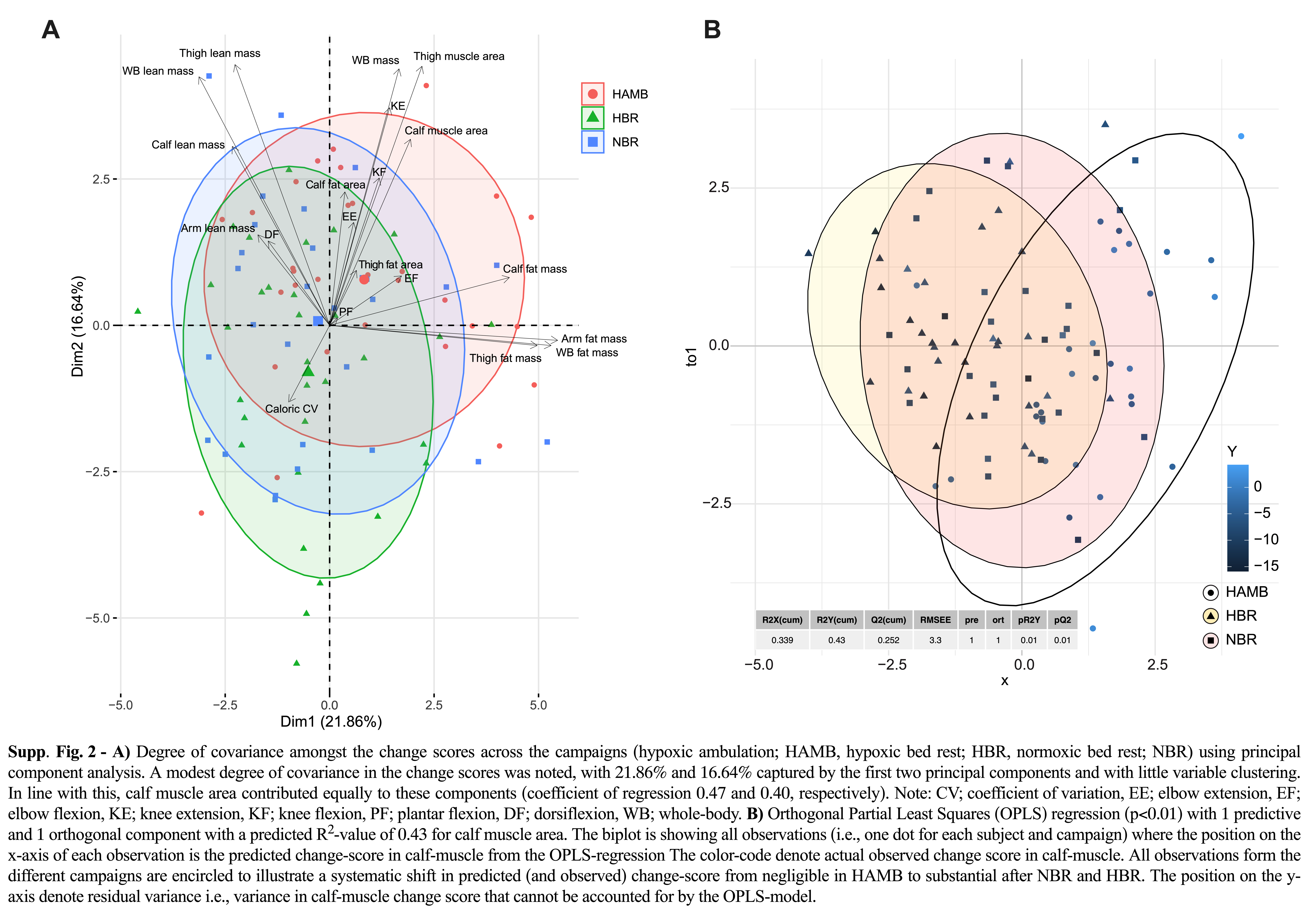

Supplement: Supplementary file 2 [file Image_2.tiff]
